# Hypertension Prevalence among Hispanics/Latinos of Dominican Background: A Transnational Comparison of HCHS/SOL and ENPREFAR-HAS-17

**DOI:** 10.5334/gh.1352

**Published:** 2024-08-26

**Authors:** Luisa Alvarez, Ayana April-Sanders, Priscilla Duran Luciano, Un Jung Lee, Katrina Swett, Cesar Herrera, Donaldo Collado, Robert Kaplan, Franklyn Gonzalez II, Martha Daviglus, Olga Garcia-Bedoya, Tali Elfassy, Neil Schneiderman, Krista Perreira, Gregory A. Talavera, Leonor Corsino, Carlos J. Rodriguez

**Affiliations:** 1 Albert Einstein College of Medicine. Department of Cardiology. Clinical Cardiovascular Research Center. 1300 Morris Park Avenue, Bronx, NY 10461, USA; 2 Rutgers School of Public Health. Department of Biostatistics & Epidemiology. 683 Hoes Lane West, Piscataway, NJ 08854, USA; 3 Northwell Health. Biostatistics Unit. 1111 Marcus Avenue Ste 107. New Hyde Park NY 11042, USA; 4 Advanced Medicine and Telemedicine Diagnostic Center [Centro de Diagnóstico y Medicina Avanzada y Telemedicina (CEDIMAT)]. Department of Cardiology. Pepillo Salcedo Street and Arturo Logroño Street, Ensanche La Fe, Santo Domingo, Dominican Republic; 5 Dominican Society of Cardiology [Sociedad Dominicana de Cardiología (SODOCARDIO)], 403 Santiago Street, Santo Domingo, Dominican Republic; 6 Albert Einstein College of Medicine. Department of Epidemiology & Population Health. 1300 Morris Park Avenue, Bronx, NY 10461, USA; 7 Fred Hutchinson Cancer Center. Public Health Sciences Division. 1100 Fairview Ave N, Seattle, WA 98109, USA; 8 University of North Carolina. Department of Biostatistics. 123 W. Franklin St., Suite 450, CB# 8030, Chapel Hill, NC 27516, USA; 9 University of Illinois at Chicago, College of Medicine. Institute for Minority Health Research. 835 S Wolcott Ave (Bldg 935). Mailbox #23 (M/C 769). Chicago, IL 60612, USA; 10 University of Miami, Miller School of Medicine. Department of Medicine. 1120 NW 14th St, Ste 822, Miami, FL 33136, USA; 11 University of Miami, Miller School of Medicine. Department of Psychology. 1120 NW 14th St, Ste 822, Miami, FL 33136, USA; 12 University of North Carolina. Department of Social Medicine. 123 W. Franklin St., CB#7240, Chapel Hill, NC 27516, USA; 13 San Diego State University. Department of Psychology. South Bay Latino Research Center. 780 Bay Blvd, Suite 200, Chula Vista, CA 91910, USA; 14 Duke University. Division of Endocrinology, Box 3451, Durham, NC 27710, USA

**Keywords:** Hypertension, Dominican Background, Cardiovascular Disease

## Abstract

**Background:**

Hispanics/Latinos of Dominican background living in United States (US) have the highest hypertension prevalence compared with other Hispanic/Latino persons.

**Objective:**

To understand cardiovascular health among Dominicans, we evaluated hypertension prevalence and risk factors among Dominicans from the US and Dominican Republic (DR) using data from Hispanic Community Health Study/ Study of Latinos [HCHS/SOL] and the Prevalencia de Hipertension Arterial y Factores de Riesgo Cardiovasculares en la República Dominicana al 2017 (ENPREFAR-HAS 17) study.

**Methods:**

Hypertension was defined as blood pressure ≥140/90 mmHg, self-reported hypertension, or antihypertensive use. Exposures included sociodemographic/socioeconomic, clinical, and lifestyle/behavioral characteristics. Weighted generalized linear models were used to estimate associations between study characteristics and hypertension prevalence (PR = prevalence ratio), age-and-sex adjusted. HCHS/SOL (n = 1,473, US Dominicans; mean age 41 years, 60.4% female) was analyzed with survey procedures, while ENPREFAR-HAS 17 (n = 2,015 DR Dominicans; mean age 40 years, 50.3% female) was analyzed with statistical analyses for simple random sampling.

**Results:**

Hypertension prevalence was 30.5% and 26.9% for DR and US Dominicans, respectively. Hypertension control was low in both cohorts (36.0% DR, 35.0% US). Alcohol use among DR Dominicans was inversely associated with hypertension prevalence (PR_DR_ = 0.8) with no association among US Dominicans. In both settings, diabetes (PR_DR_ = 1.4; PR_US_ = 1.4) and obesity (PR_DR_ = 1.8; PR_US_ = 2.0) were associated with greater hypertension prevalence in Hispanics/Latinos of Dominican background. Physical activity was lower among US Dominicans (PR = 0.80) but higher among DR Dominicans (PR = 1.16); all p < 0.05.

**Conclusions:**

Variations in social, lifestyle/behavioral, and clinical characteristics associated with hypertension among Dominicans in the US and DR were identified, suggesting that social context and cultural factors matter among immigrant populations.

## List of Abbreviations

**Table:** 


ABBREVIATION	FULL DESCRIPTION

US	United States

DR	Dominican Republic

HCHS/SOL	Hispanic Community Health Study/ Study Of Latinos

ENPREFAR-HAS 17	Encuesta Prevalencia De Hipertensión Arterial Sistémica y Factores De Riesgo Cardiovasculares En La República Dominicana al 2017

HTN	Hypertension

PR	Prevalence Ratio

CI	Confidence Intervals

SE	Standard Error

CVD	Cardiovascular Disease

HLDB	Hispanics/Latinos of Dominican Background

BP	Blood Pressure

DR Dominicans	Dominicans Living In The DR

SODOCARDIO	Sociedad Dominicana De Cardiología

BMI	Body Mass Index

US Dominicans	Hispanics/Latinos of Dominican Background Living in the US

JNC7	Seventh Report of The Joint National Committee On Prevention, Detection, Evaluation, And Treatment Of High Blood Pressure

ACC/AHA	American College of Cardiology/American Heart Association

HS	High School

GPAQ	Global Physical Activity Questionnaire

GLM	Generalized Linear Models

SASH	Short Acculturation Scale for Hispanics

MESA	Multiethnic Study of Atherosclerosis

CARMELA	Cardiovascular Risk Factor Multiple Evaluation in Latin America

NHLBI	National Heart, Lung, And Blood Institute


## Introduction

Hispanic/Latino populations are particularly vulnerable to cardiovascular disease (CVD), a leading cause of mortality in the United States (US) and worldwide (1,2,3). Although hypertension is the most preventable and modifiable risk factor for CVD, Hispanic/Latino adults in the US are more likely than non-Hispanic whites to have undiagnosed, untreated, or uncontrolled hypertension (4,5). Emerging data from the Hispanic Community Health Study/Study of Latinos (HCHS/SOL), one of the largest epidemiologic studies of Hispanics/Latinos in the US, showed that Hispanics/Latinos of Dominican background (HLDB) living in the US have the highest hypertension prevalence when compared to Hispanics/Latinos of other backgrounds (6,7). These results were consistent in other population-based studies where Dominican participants had higher hypertension rates than non-Hispanic whites and other Hispanic/Latino backgrounds (8,9,10,11). It is evident that there is heterogeneity within Hispanic/Latino backgrounds that influences disease prevalence, suggesting that CVD risk factors may not be generalizable to Hispanics/Latinos in aggregate (3). HLDB living in the US represent one of the fastest-growing US Hispanic/Latino backgrounds and remain the fifth largest Hispanic/Latino population in the US (12). There is a paucity of studies analyzing health outcomes between US-born Dominicans and foreign-born Dominicans. Thus, a transnational comparison of hypertension prevalence between HLDB in the US and Dominicans in the Dominican Republic (DR) could help elucidate similarities and differences in disease burden, modifiable risk factors, and management strategies across countries.

The present study examined hypertension prevalence in Hispanic/Latino adults of Dominican background (US-born and foreign-born) living in the US who were enrolled in HCHS/SOL and in Dominican adults living in the DR who were enrolled in the Encuesta [EN] Prevalencia [PRE] de Hipertensión Arterial Sistémica [HAS] y Factores de Riesgo [FAR] Cardiovasculares en la República Dominicana al 2017 (ENPREFAR-HAS 17) study. We examined the impact of sociodemographic/socioeconomic, clinical, and lifestyle/behavioral risk factors, along with acculturation, on hypertension prevalence among HLDB from two social contexts—the US and the DR.

## Methods

### Study population

Hypertension among HLDB living in the US was assessed using data from the Hispanic Community Health Study/Study of Latinos (HCHS/SOL), a community-based prospective cohort study of 16,415 self-identified Hispanic/Latino persons designed to examine risk and protective factors for chronic diseases among Hispanic/Latino adults in the US. The study sampling strategy (13) and implementation (14) are detailed elsewhere. In brief, using a stratified two-stage probability sampling strategy, Spanish and/or English-speaking Hispanic/Latino adults aged 18–74 years and residing in four US metropolitan cities (Chicago, IL; San Diego, CA; New York, NY; and Miami, FL), were recruited to participate in HCHS/SOL (13).

HCHS/SOL enrolled a representative sample of Hispanics/Latinos from Cuban, Dominican (n = 1,473, 9%), Mexican, Puerto Rican, Central American, and South American backgrounds at baseline (Visit 1, 2008–2011). HLDB enrolled in HCHS/SOL are predominantly from the Bronx, NY, accounting for 94.4% of the total sample of HLDB. The remaining HLDB in HCHS/SOL were recruited from Miami, FL (4.4%), Chicago, IL (0.8%), and San Diego, CA (0.3%). Clinical and epidemiologic data were collected during baseline and follow-up visits (Visit 2, 2014–2017), but only Visit 1 data were analyzed in this study. Participants underwent comprehensive clinical examinations, including assessment of CVD risk factors and blood samples collection; information on sociodemographic/socioeconomic, lifestyle factors, acculturation, and medical history was collected by questionnaires. The study was approved by the Institutional Review Boards at each site where all subjects gave written consent.

Hypertension among Dominicans living in the DR [DR Dominicans] was assessed using the ENPREFAR-HAS 17 national survey (15). ENPREFAR-HAS 17 was a national survey funded by the Dominican government and conducted by the Dominican Society of Cardiology (Sociedad Dominicana de Cardiología, SODOCARDIO) in 2017 to determine hypertension prevalence and CVD risk factors among adults in the DR. Participants in ENPREFAR-HAS 17 were recruited from nine public health geographical regions of the DR, including urban and rural areas in each region, to obtain a representative sample of the entire country. Randomly selected household members from each designated area were interviewed door-to-door between May 13 and June 25, 2017. There were only interviews and no clinical visits in this study. Participants were selected according to sex and age quotas established in the 2010 Dominican Census. During the interviews, a close-questioned survey was conducted for each participant. BP and individual anthropometric measures (height, weight, body mass index [BMI], waist and hip circumferences) were recorded. This resulted in a representative sample (N = 2,015) of Dominicans aged 18–102 years, all of whom were included in these analyses. The data set excluded pregnant women and participants with chronic disabling diseases, such as cancer or liver cirrhosis.

### Blood pressure and hypertension assessment

In HCHS/SOL, systolic and diastolic BPs were averages of three consecutive readings taken during Visit 1 with participants positioned seated using the Omron HEM 907XL sphygmomanometer (13). Pulse and BP were measured in the right arm of all HCHS/SOL participants. In ENPREFAR-HAS 17, participants had their systolic and diastolic BPs measured in two consecutive readings, 5 minutes apart, using a Riester aneroid sphygmomanometer on their dominant arm. The second measurement was recorded as the definitive BP reading for ENPREFAR-HAS 17, a measurement approach reflective of real-world clinical practice in the DR (15).

The Seventh Report of the Joint National Committee on Prevention, Detection, Evaluation, and Treatment of High Blood Pressure (JNC7) guidelines (16) were used in both HCHS/SOL and ENPREFAR-HAS 17 to define hypertension as systolic BP ≥ 140 mmHg or diastolic BP ≥ 90 mmHg. Self-reported use of antihypertensive medications was also employed to classify hypertension in both cohorts. JNC7 were the latest hypertension guidelines available and contemporaneous at the time of HCHS/SOL visit 1 (2008–2011) and were also used in ENPREFAR-HAS 17. As a secondary analysis, we defined hypertension based on the most current hypertension guidelines for overall contrast of hypertension prevalence, which are outlined as follows by the American College of Cardiology/American Heart Association (ACC/AHA) (17): systolic BP ≥ 130 mmHg, or diastolic BP ≥ 80 mmHg.

To further assess hypertension, we defined hypertension awareness, treatment, and control. Hypertension awareness was defined in both cohorts as hypertensive participants who confirmed they were informed by a doctor of their hypertension status (HCHS/SOL) or participants who self-identified as hypertensive (ENPREFAR-HAS 17). Hypertension treatment was defined in both cohorts as answering yes to questions regarding whether they take antihypertensive medication. The JNC7 threshold (<140/90 mmHg, controlled vs. ≥140/90 mmHg, uncontrolled) was used in both cohorts to define hypertension control.

### Socioeconomic, clinical, and lifestyle/behavioral risk factors

#### Socioeconomic risk factors

Hypertension prevalence was analyzed according to categories of age (years), sex (male/female), and educational level (as a proxy for socioeconomic status [SES]: less than high school [HS], HS or equivalent, greater than HS). No income data was available in ENPREFAR-HAS 17, hence income was excluded for SES.

#### Clinical risk factors

Clinical factors included BMI (measured as kg/m^2^ in both cohorts) and diabetes status. Past medical history of diabetes was defined based on three criteria for HCHS/SOL participants:

1. self-reported diabetes diagnosis.
2. self-reported diabetic medication use.
3. laboratory-tested fasting plasma glucose ≥126 mg/dL, non-fasting plasma glucose of > 200 mg/dL, 2-hour oral glucose tolerance test > 200 mg/dL, or glycosylated hemoglobin (HbA1c) > 6.5%.

HCHS/SOL participants were classified as having normal glucose tolerance, prediabetes, or diabetes. Participants in ENPREFAR-HAS 17 were categorized as having no diabetes or diabetes based on self-reported diabetes history (yes/no) and self-report of medication use.

#### Lifestyle/behavioral risk factors

Lifestyle and behavioral factors included cigarette use (current and former/never smokers), alcohol use (current, former, and never users), and physical activity. Physical activity was assessed in HCHS/SOL through the Global Physical Activity Questionnaire (GPAQ). In ENPREFAR-HAS 17, measurement of physical activity was based on self-identification as active or inactive (yes/no), as well as measures of frequency and timing for those self-reporting to be active.

#### Study variables harmonization

When analyzing data from HCHS/SOL and ENPREFAR- HAS 17, several variables were re-coded to facilitate comparability between the two cohorts. To harmonize the variables for both cohorts, measurements such as diabetes, alcohol use, physical activity, BMI, and education were modified. For the diabetes measurements in HCHS/SOL, the normal glucose tolerance and pre-diabetes groups were combined into a single ‘no diabetes’ category to match ENPREFAR-HAS 17 variable coding. Similarly, for measures of alcohol use, in HCHS/SOL, the alcohol use categories of former and never were combined into a single ‘no alcohol use’ category to match the dichotomous alcohol use variable coding in ENPREFAR-HAS 17. In HCHS/SOL, physical activity is divided into inactivity, low activity, medium activity, and high activity based on the GPAQ total physical activity in minutes/day, while ENPREFAR-HAS 17 has two categories: active and not active. The low, medium, and high activity categories of HCHS/SOL were combined into a single ‘active’ category to harmonize datasets. BMI outputs in ENPREFAR-HAS 17 included underweight, normal, overweight, and obese categories based on clinical guidelines. The underweight and normal weight BMI categories were combined to form a <25 kg/m^2^ category for comparison with HCHS/SOL. The ENPREFAR-HAS 17 variable for education was re-coded to combine ‘no education’ and ‘less than HS’ categories to form a single ‘less than HS’ category for comparison with HCHS/SOL.

### Statistical analysis

Descriptive analyses were performed to estimate hypertension prevalence and sociodemographic/socioeconomic, clinical, and lifestyle characteristics distribution within each cohort. The HCHS/SOL and ENPREFAR-HAS 17 samples were analyzed separately. Median interquartile range (IQR) was calculated for continuous variables and proportions (%) for categorical variables. Unadjusted analyses were performed using linear regression models for continuous variables and *χ*^2^ tests for categorical variables.

Generalized linear models (GLM) were used to test the association between sociodemographic/socioeconomic, clinical, and lifestyle/behavioral risk factors and hypertension prevalence separately in each cohort and compared (18). We calculated prevalence ratios (PR) and 95% confidence intervals (CI) with Poisson regression models with robust standard error (SE) fit to the binary response using the GLM procedure (18). We used standard procedures to ensure that our models fit appropriately to the assumptions of a Poisson regression with binary distribution. The PR is preferable to the odds ratios when the outcome is common and allows for better interpretability, among other favorable statistical properties (18). Models are presented unadjusted and adjusted for age and sex.

Several sensitivity analyses were conducted. First, using stratified analyses, we explored differences in the characteristics between US-mainland and foreign-born HLDB within the HCHS/SOL sample and compared them to ENPREFAR-HAS 17. We stratified the US Dominican HCHS/SOL sample into two groups based on nativity status, US-born and foreign-born (DR-Born) Dominicans, and excluded participants born in countries other than DR and the US (N = 30). Place of birth and nativity data were unavailable in the ENPREFAR-HAS 17 database. Second, we harmonized BP in HCHS/SOL to the ENPREFAR-HAS 17 approach by taking the second BP measurement as definitive. We then reran all the analyses to determine the unadjusted and age-and-sex adjusted prevalence ratios of hypertension prevalence (defined by JNC7 and ACC/AHA) according to sociodemographic/socioeconomic, clinical, and lifestyle/behavioral risk factors. Lastly, for the impact of acculturation on hypertension prevalence in HLDB living in the US, we included measures of acculturation (language preference [English/Spanish]), years in the US (≥10 years and <10 years), and the Short Acculturation Scale for Hispanics (SASH) [language and ethnic social relations subscales] for foreign-born HLDB from the HCHS/SOL cohort (19). Higher SASH scores represent greater orientation to the non-Latino US culture.

**Figure 1:**
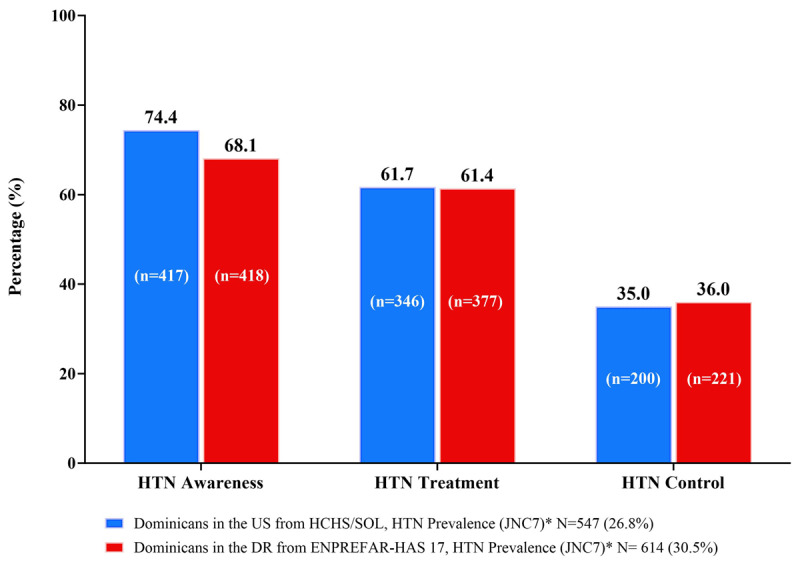
Hypertension^1^ (HTN) awareness, treatment, and control among US Dominicans (HCHS/SOL) and DR Dominicans (ENPREFAR-HAS 17). ^1^Hypertension is defined using JNC7 guidelines: systolic blood pressure ≥ 140 mmHg, or diastolic blood pressure ≥ 90 mmHg. All Ns are unweighted, but HCHS/SOL proportions (%) are weighted.

**Figure 2:**
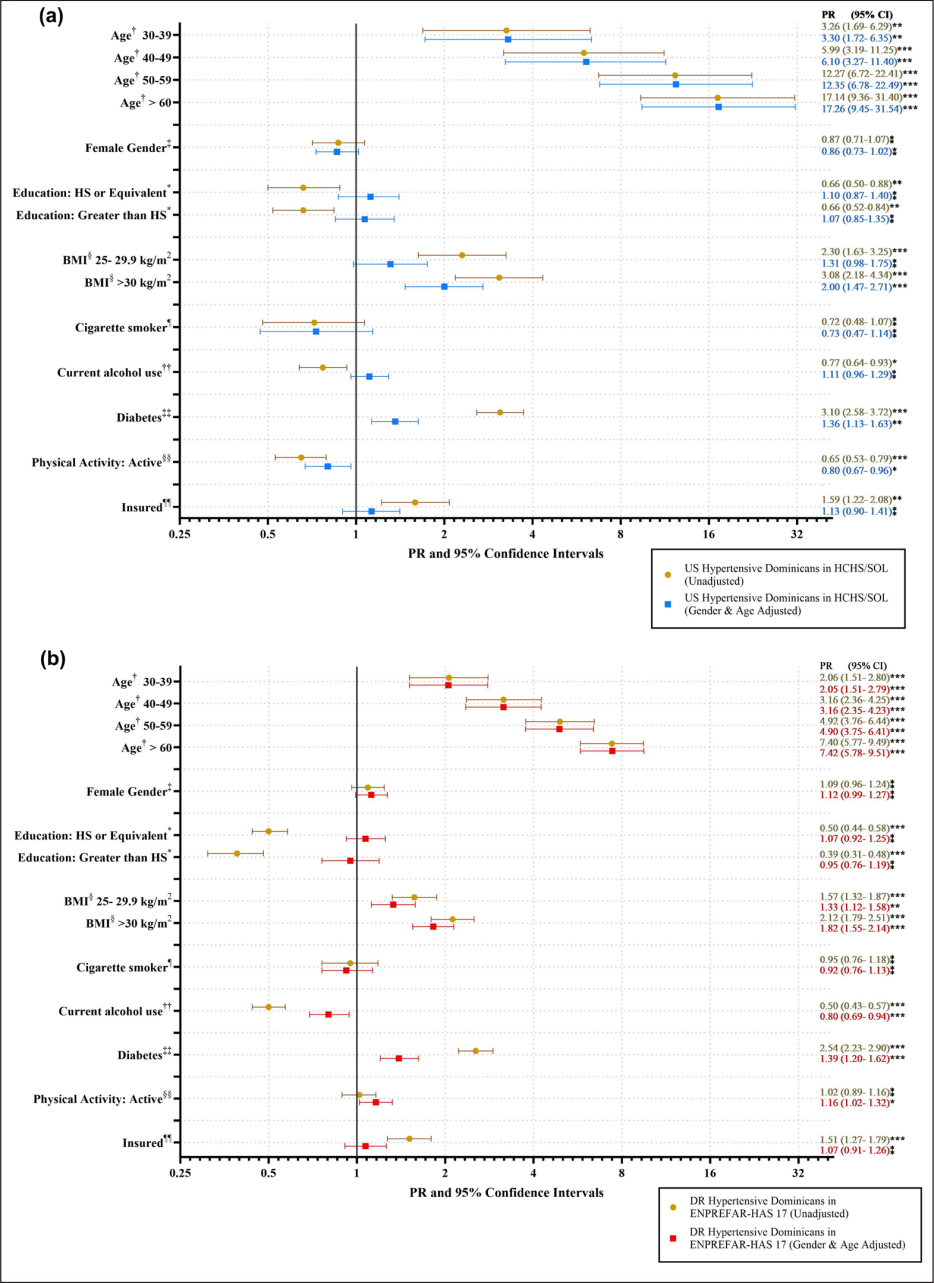
Unadjusted and adjusted hypertension^1^ prevalence ratios among US Dominicans (HCHS/SOL^2^) and DR Dominicans (ENPREFAR-HAS 17). **(a)** shows unadjusted and age-and-gender adjusted PR among Dominicans in the US from the HCHS/SOL cohort. **(b)** shows unadjusted and age-and-gender adjusted PR among Dominicans in the DR from the ENPREFAR-HAS 17 cohort.^1^Hypertension is defined using JNC7 guidelines: systolic blood pressure ≥ 140 mmHg, or diastolic blood pressure ≥ 90 mmHg. Prevalence ratios for prevalent hypertension defined per ACC/AHA guidelines can be found in Supplemental Table 1;^2^ Weighted models; †Not adjusted for age in age/gender adjusted models. Reference value: Age 18–29 years old; ‡Not adjusted for sex in age/gender adjusted models. Reference value: Male Gender; *Reference Value: Less than HS (high school); §Reference Value: BMI: <25 kg/m^2^; ¶ Reference Value: Non-smoker [cigarettes]; ††Reference Value: Former/Never (Alcohol consumer); ‡‡Reference Value: No diabetes; §§Reference Value: Inactive (physical activity); ¶¶ Reference Value: No health insurance. P values are reported as follows: * < 0.05, ** < 0.01, *** < 0.0001, ⁑ > 0.05.

Survey procedures using sampling weights in SAS 9.4 (SAS Institute, Gary, NC) were performed to account for the complex survey design and clustering for HCHS/SOL data, which differs from the simple random sampling of ENPREFAR-HAS 17 data. ENPREFAR-HAS 17 data were analyzed by using standard statistical analyses. All statistical tests were two-sided at a significance level of 0.05. Figures 1 and 2 (a and b) were created using GraphPad Prism version 8.0.0 for Windows.

## Results

### Sample demographics

Sociodemographic/socioeconomic, lifestyle, and clinical characteristics are presented in Table 1. Median IQR ranges for age were similar among US Dominicans (38.1 years) and DR Dominicans (37.0 years). The HCHS/SOL sample had a slightly higher proportion of US Dominican females (61%) than DR Dominican females (50%) from the ENPREFAR-HAS 17 sample. The proportion of those with health insurance was similar across both cohorts. US Dominicans had lower educational levels than DR Dominicans, with 37% vs. 28% reporting less than a high school education.

**Table 1:** Baseline characteristics of the study cohorts, adult Hispanics/Latinos of Dominican Background living in the US (HCHS/SOL, 2008–2011) and in DR (ENPREFAR-HAS 17, 2017).


	US DOMINICANS (HCHS/SOL3) N = 1473	DR DOMINICANS (ENPREFAR-HAS 17) N = 2015

**Age, years, median IQR (Q1, Q3)**	38.1 (25.1, 50.1)	37.0 (26.0, 52.0)

**Age (years) categories, n (%)**		

**18–29**	297 (32.9)	662 (34.0)

**30–39**	174 (18.7)	420 (21.6)

**40–49**	375 (21.4)	300 (15.4)

**50–59**	391 (16.0)	271 (13.9)

**60+**	236 (11.1)	294 (15.1)

**Gender, n (%)**		

**Female**	963 (60.4)	1013 (50.3)

**Male**	510 (39.5)	1002 (49.7)

**Education level, n (%)**		

**Less than HS**	616 (36.8)	569 (28.2)

**HS or Equivalent**	303 (23.5)	1019 (50.6)

**Greater than HS**	554 (39.6)	427 (21.2)

**Health insurance, n (%)**		

**Insured**	1040 (70.8)	1459 (72.4)

**Uninsured**	365 (29.2)	556 (27.6)

**Hypertension status (JNC7)1, n (%)**		

**Hypertensive**	547 (26.8)	614 (30.5)

**Normotensive**	926 (73.2)	1401 (69.5)

**Hypertension status (ACC/AHA)2, n (%)**		

**Hypertensive**	771(43.4)	1,113 (55.2)

**Normotensive**	702 (56.6)	902 (44.8)

**BMI, kg/m** ^2^ **, n (%)**		

**<25**	296 (22.3)	790 (39.2)

**25–29.9**	564 (39.9)	668 (33.1)

**30+**	604 (40.8)	553 (27.4)

**Diabetes status, n (%)**		

**No Diabetes**	1190 (84.7)	1886 (93.6)

**Diabetes**	283 (15.3)	129 (6.4)

**Cigarette use, n (%)**		

**Non-smoker**	1317 (88.5)	1791 (88.9)

**Current**	155 (11.5)	224 (11.1)

**Alcohol use, n (%)**		

**Former/ Never**	721 (44.6)	916 (45.5)

**Current**	751 (55.4)	1099 (54.5)

**Physical activity, n (%)**		

**Inactive**	294 (18.1)	1209 (60.0)

**Active**	1172 (81.9)	806 (40.0)


^1^ JNC7 hypertension definition: systolic blood pressure ≥ 140 mmHg, or diastolic blood pressure ≥ 90 mmHg ^2^ ACC/AHA hypertension definition: Systolic blood pressure ≥ 130 mmHg, or diastolic blood pressure ≥ 80 mmHg. ^3^ Weighted statistics. Note: All Ns are unweighted, but HCHS/SOL proportions (%) and means are weighted.

Hypertension prevalence varied by BP guidelines. Among US Dominicans, hypertension prevalence was 27% (JNC7 criteria) and 43% (ACC/AHA criteria). Hypertension was more prevalent among DR Dominicans: 31% (JNC7 criteria) and 55% (ACC/AHA criteria). A greater proportion of US Dominicans were obese (BMI > 30 kg/m^2^) and had diabetes (40% obese, 15% diabetes) compared to DR Dominicans (27% obese, 6% diabetes). As for lifestyle/behavioral characteristics, US Dominicans reported more engagement in physical activity (82%) than DR Dominicans (40%). Current cigarette and alcohol use were similar in both cohorts.

### Hypertension awareness, treatment, and control

Hypertension prevalence among DR Dominicans was slightly higher (31%) than among US Dominicans (27%). Among those with hypertension (Figure 1), awareness was common (74% US Dominicans, 68% DR Dominicans); and most were receiving antihypertensive treatment (62% US Dominicans, 61% DR Dominicans), but overall, hypertension control was low in both groups (35% US Dominicans; 36% DR Dominicans).

### Hypertension prevalence ratios

Figure 2 shows PR for associations between sociodemographic/socioeconomic, clinical, and lifestyle/behavioral risk factors and prevalent hypertension using the JNC7 criteria among US and DR Dominicans in unadjusted and age- and sex-adjusted models. In both cohorts, hypertension prevalence increased as age increased, but results indicated higher PRs for US Dominicans compared to DR Dominicans with increasing age. For example, in adjusted models [Figure 2(a)], US Dominicans 50–59 years old were 12 times more likely to have prevalent hypertension compared with 18–29 years old (95% CI: 6.78–22.49), while DR Dominicans 50–59 years old [Figure 2(b)] were 4.9 times more likely to have prevalent hypertension than 18–29 years old (95% CI: 3.75–6.41). Linear regression was used to test if age significantly predicted hypertension prevalence. Age significantly predicted hypertension prevalence among US Dominicans in both unadjusted (β = 0.05, p-value .0001) and adjusted (β = 0.06, p-value .0001) models. The same relationship was found among DR Dominicans, in which age significantly predicted hypertension prevalence in both unadjusted (β = 0.04, p-value .0001) and adjusted (β = 0.03, p-value .0001) models.

In unadjusted models, greater education was associated with lower hypertension prevalence for both cohorts. Still, the protective effects diminished in adjusted models and were no longer significant [Figure 2(a) and 2(b)]. Obesity (BMI > 30 kg/m^2^) (PR_US_ = 2.00, 95% CI: 1.47–2.7; PR_DR_ = 1.82, 95% CI: 1.55–2.14) and diabetes (PR_US_ = 1.36, 95% CI: 1.13–1.63; PR_DR_ = 1.39, 95% CI: 1.20–1.62) were significant predictors of prevalent hypertension among both US and DR Dominicans in adjusted models, having had some attenuation from unadjusted models. The association between hypertension prevalence and lifestyle characteristics varied by cohort. Alcohol use was inversely associated with hypertension prevalence for DR Dominicans (PR_DR_ = 0.80, 95% CI: 0.69–0.94), with no association among US Dominicans (PR_US_ = 1.11, 95% CI: 0.96–1.29) in adjusted models. Conversely, physical activity was protective against prevalent hypertension among US Dominicans (PR_US_ = 0.8, 95% CI: 0.67–0.96) but associated with increased hypertension prevalence among DR Dominicans (PR_DR_ = 1.16, 95% CI: 1.02–1.32) in adjusted models.

The results using the ACC/AHA criteria for hypertension shown in Supplemental Table 1 were consistent with the JNC7 criteria presented in Figure 2.

### Sensitivity analysis

#### Nativity status

Supplemental Table 2 shows that among those living in the US, US-Born Dominicans were younger (mean age 23 years) with lower hypertension prevalence (3%, JNC7 and 18%, ACC/AHA) than DR-Born Dominicans, who were older (mean age 42 years) and had a higher hypertension prevalence (31%, JNC7 and 49%, ACC/AHA).

#### Blood pressure harmonization

We harmonized BP values in HCHS/SOL to the ENPREFAR-HAS 17 approach by taking the second BP measurement as definitive. Findings using this approach (Supplemental Tables 3–4) were similar to the main analysis (Figure 2) assessing factors associated with hypertension prevalence (JNC7 and ACC/AHA). We also harmonized BP values in the Dominican subset in HCHS/SOL per nativity status, US-Born and DR-Born Dominicans (Supplemental Tables 3–4). However, due to the small sample size of US-born Dominicans (n = 138) and low prevalence of hypertension among this group, PRs for the nativity-stratified harmonized analysis were obtained only for the DR-born Dominicans residing in the US. Again, the BP harmonized analysis results were consistent with the main analysis when stratified by US nativity status.

#### Acculturation measures in Hispanics/Latinos of Dominican background (HLDB) living in the US

Lastly, we analyzed the association of hypertension prevalence, defined by JNC7 and ACC/AHA (Supplemental Tables 5–6), with measures of acculturation in HLDB from the HCHS/SOL cohort. Results showed that shorter time in the US (PR = 0.71, 95% CI: 0.54–0.91), higher median SASH language (per unit SASH score, PR = 0.39, 95% CI: 0.30–0.51) and SASH social (PR = 0.68, 95% CI: 0.57–0.80) subscale scores were associated with lower prevalent hypertension; while Spanish language preference (PR = 3.36, 95% CI: 2.09–5.40) and being foreign-born (PR = 6.31, 95% CI: 3.06–13.0) were associated with greater hypertension prevalence. Despite these results, no statistical significance was found after age-and-sex adjustments.

## Discussion

We estimated hypertension prevalence among HLDB using two samples of adults, those living in the US (urban settings) and those living in the DR (urban and rural settings). This is the first study to compare hypertension prevalence among Dominicans from two social contexts. Based on JNC7 and ACC/AHA criteria, hypertension prevalence among US Dominicans ranged from 27% to 44%, respectively, whereas hypertension prevalence among DR Dominicans ranged from 31% to 55%. We also showed that HLDB, whether living in the US or the DR, shared common risk factors for hypertension, including obesity and diabetes. In contrast, physical activity was lower among hypertensive US Dominicans but was associated with increased hypertension among DR Dominicans. Reported alcohol use was associated with reduced hypertension prevalence, but only among DR Dominicans.

Our findings align with existing research that identified greater hypertension prevalence among Hispanic/Latino persons of Caribbean background (7). Although Hispanic/Latino persons are often aggregated as a single ethnic group, considerable heterogeneity in cultural, socioeconomic, and genetic factors exists between the different ancestral groups and may influence hypertension prevalence, treatment, and control. In HCHS/SOL, age-standardized hypertension prevalence was found to be highest among Hispanic/Latino persons of Caribbean background: Dominicans (men, 33%; women, 26%), Cubans (men, 29%; women, 26%), Puerto Ricans (men, 27%; women, 29%), and Central Americans (men, 25%; women, 26%), while hypertension prevalence was notably lower among Hispanics/Latinos of Mexican (men, 21%; women, 20%) and South American (men, 20%; women, 16%) backgrounds (20). Similarly, the Multiethnic Study of Atherosclerosis (MESA) showed a significantly higher hypertension prevalence among Dominican Americans (53%) and Puerto Rican Americans (43%) compared with Mexican Americans (38%) (21).

Observations of Hispanic/Latino persons living outside of the US have also shown similar variations in hypertension prevalence. Hypertension prevalence among seven Latin American cities approximated US averages (14–27%) in the Cardiovascular Risk Factor Multiple Evaluation in Latin America (CARMELA) study (22). However, markedly lower hypertension prevalence was observed among US Hispanics/Latinos in some city centers, like Mexico City, where the prevalence was 12% (22). The results of this study and previously reported data further support the efforts to disaggregate data for Hispanic/Latino backgrounds to accurately reflect sociocultural impacts on hypertension rates and CVD within the Hispanic/Latino community.

Our study suggests that even within a particular cultural or ancestral group, there may be maintenance of native sociocultural lifestyles and behaviors that continue to drive certain disease outcomes. According to our results, HLDB living in the US share some of the same CVD factors for hypertension as those living in the DR, including a higher BMI and diabetes. The Dallas Heart Study, a cross-sectional multiethnic population-based study of Dallas (Texas) residents, found that factors such as BMI and diabetes were only partially responsible for the relationship of acculturation to hypertension among Hispanics/Latinos (23). We hypothesize that US Dominicans may retain aspects of their native socio-cultural lifestyles (transnational identity) such as dietary habits, which might predispose them to the development of comorbidities (like obesity and diabetes) in the same way it does in the DR. The maintenance of this transnational identity might be attributed to a variety of factors, including circular migration, population settlement in localized areas of the US (with a predominantly Dominican population), retaining contact with relatives and friends in the DR, dual citizenship, and close economic ties to the DR through migrant remittances (24,25,26). In the book ‘Quisqueya on the Hudson: The Transnational Identity of Dominicans in Washington Heights,’ it is argued that Dominicans in the US are attempting to recreate the life they lived on the island and maintain their native cultural atmosphere (traditional foodways, spoken language, music taste, shopping preferences, and religious affiliation) to connect their country of origin and their country of settlement (25). Other factors considered as part of this transnational identity include dietary and culinary habits. The presence of small independent Dominican grocery stores known as *bodegas* in New York City, but prevalent throughout Dominican population-based areas of the US, are known for selling Caribbean and Latin American food ingredients commonly used in Dominican cuisine, signaling how Dominicans in the US continue to cook and consume traditional dishes from their native country (25). Thus, maintaining the same dietary habits from the DR could predispose Dominicans to obesity, diabetes, and hypertension. To further support or refute this hypothesis, more research is needed on the associations between diet and culture in individuals of Dominican backgrounds.

Alternatively, we cannot ignore the possibility that the transnational identity among Dominicans may be influenced by elements from US cultural involvement as surrogates for acculturation—like time spent living in the US, number of friends who had lived in the US, English proficiency, and use of US electronic media and language (26,27). A modified version of this transnational identity incorporating US cultural elements might especially be seen in second-generation Dominicans in the US (25), which could partly explain the differences found in other measures of sociodemographic/socioeconomic, lifestyle/behavioral, and clinical characteristics among our two cohorts of Dominican populations.

Some of our findings were inconsistent with past literature. Alcohol use was found to be protective against hypertension prevalence in DR Dominicans, which differs from previous evidence (28). In a recent review article, it was noted that intervention studies and Mendelian randomization analyses confirm that the relationship between greater alcohol consumption and higher BP is causal (21). Additionally, the concept that low alcohol consumption reduced BP, particularly in women, is an unstable finding and likely not representative of a true causal relationship (29). The review article also summarized current evidence placing alcohol-related hypertension in the causal pathway between alcohol use and increased risk for cardiovascular outcomes. One possible explanation for the paradoxical findings of alcohol use and reduced hypertension prevalence in DR Dominicans in our study could be linked to how alcohol use was captured in ENPREFAR-HAS 17. Drinking patterns, type of alcoholic drinks, and amount of alcohol consumed were not captured, and it may be problematic to compare those who consume alcohol to subjects who do not. For studies that overestimate the benefits of alcohol, nondrinkers are regarded by some authors (30) as an inappropriate comparison group because unmeasured and uncontrolled confounding could include dietary and lifestyle differences that could affect the complex relationship between alcohol use and cardiovascular risk factors. Alcohol consumption patterns, for example, are likely to impact a wide range of disease processes in a way that nondrinkers are not affected because they are not exposed to these external factors.

Physical activity is often cited as cardioprotective exposure and is linked with significantly benefiting cardiovascular health. Our finding of reduced hypertension prevalence among US Dominicans reporting physical activity is consistent with the literature (31), but interestingly, we observed a direct association between physical activity and hypertension prevalence among DR Dominicans. We expected to see consistent results among both groups for physical activity because the physical activity patterns among Dominicans seem to stay consistent between those living in the US and those in DR. As noted by some authors (32), HLDB in HCHS/SOL engage in more regular physical activity related to work (occupational-related physical activity) and transportation (active transport) than other Hispanic/Latino persons. This finding is attributed to the fact that most HLDB in HCHS/SOL were recruited from the Bronx, NY, a city with widely accessible public transportation used by HLDB for their daily work commutes. A similar relationship can be found between physical activity and active transport in Latin American and Caribbean countries such as the DR, in which physical activity is also predominantly occupational. However, as opposed to HLDB in the US, where occupational and active transport physical activity is a choice, occupational and active transport physical activity constitutes a necessity for DR Dominicans. When private or public transportation is not economically feasible for a Dominican in the DR, walking is the best alternative mode of transportation to commute to work. The ENPREFAR-HAS 17 study used a binary response for physical activity (inactive, active), while the physical activity item used in HCHS/SOL utilized the GPAQ questionnaire that includes other factors to define physical activity such as leisure time, transportation, and work-related activity. Therefore, differences in how physical activity was measured across cohorts may explain our paradoxical findings. Additionally, there is a strong likelihood that the paradoxical findings for alcohol and physical activity could be explained by reverse causation. In other words, US Dominicans with a hypertension diagnosis could change their lifestyle upon physician advice by increasing exercise and giving up alcohol.

Lastly, there was poor hypertension control among HLDB, 35%–36%, regardless of where they lived, in the US or the DR. Recent studies have found consistently low hypertension control rates among DR Dominicans both at rural (33) and urban (34) communities. Strategies to improve hypertension and risk factor control among HLDB entail country-specific public health strategies tailored to the specific environment where HLDB are immersed. In the DR, strategies to improve hypertension should include:

1. more continued population surveillance campaigns by the public health department to identify undiagnosed hypertensives like ENPREFAR-HAS 17.
2. better country-wide access to medications and antihypertensive treatment to improve medication adherence.
3. provider-oriented educational campaigns to decrease clinical inertia on hypertension treatment.
4. population-oriented educational campaigns to promote lifestyle changes such as increased physical activity, healthy and low sodium diet, and decrease smoking and alcohol consumption.
5. culturally appropriate hypertension campaigns to mitigate the use of folkloric medicine as a sole treatment for hypertension.

In contrast, to improve hypertension prevention and control among US Dominicans, it is necessary to address healthcare barriers in the US that are prevalent among immigrant and minority groups such as language barriers, discrimination and racism, poor physician-patient trust, insufficient health literacy, decreased cultural competency among healthcare providers, and decreased inclusion in hypertension clinical trials. Nevertheless, public health strategies designed to reduce clinical inertia and ensure medication accessibility might benefit both US Dominicans and DR Dominicans alike.

### Study strengths and limitations

Our study adds valuable information about how migration and nativity are intertwined with the clinical, social, and behavioral contexts that could pose cardiovascular risks in a Hispanic/Latino community that has lately emerged with an increased risk of hypertension (6,7). A major strength of our study is the inclusion of two large community samples representing adults of Dominican background: those living in the US and those living in the DR. To the best of our knowledge, this constitutes the first study to compare hypertension prevalence between US Dominicans and DR Dominicans. Another strength of our study was the use of standardized protocols by health professionals from both cohorts to measure BP and other anthropometric measurements. A rigorous process of harmonization was performed for all variables.

There are some limitations when interpreting the findings from our study. We encountered constraints in analyzing and comparing studies of different designs. Analytically, HCHS/SOL used a probability sampling protocol to randomly select and recruit study participants from a target population of census tracts/blocks. Older adults were also over-sampled because the study’s aims focused on endpoint outcomes. Therefore, sampling weights, cluster sampling, and stratification are necessary for HCHS/SOL analyses to represent the target population. Meanwhile, ENPREFAR-HAS 17 utilized a simple random sampling design and unweighted data. While considerations were made to standardize the two datasets, interpretations would have been hindered by this data weighting issue, thus we reported prevalence and correlates separately in the two cohorts.

As a longitudinal study, HCHS/SOL was able to measure risk factors over time through cohort follow-up, while ENPREFAR-HAS 17 was a cross-sectional study with a single measure of risk factors. The cross-sectional nature of the analysis limits our ability to rule out reverse causation and results that unmeasured confounders may explain. Next, several variables had to be harmonized to improve comparability between the studies. In some instances, a measure needed to become less granular to gain harmony between the cohorts, thus resulting in a loss of information that may have explained some of the differences in our findings between cohorts.

US Dominicans from HCHS/SOL were recruited from large US cities, with the majority recruited from the New York site, which may not be representative of Hispanics/Latinos of Dominican background living in the US and therefore introduce selection bias. In contrast, the ENPREFAR-HAS 17 sample included Dominicans from both urban and rural areas in the DR, which could affect the comparability across cohorts to one extent or another. Cultural and acculturative factors were unavailable in the ENPREFAR-HAS 17 data, which limited our ability to compare and describe these details across studies and as they relate to hypertension. ENPREFAR-HAS 17 and HCHS/SOL are population-based samples with dissimilar recruitment methods, which might partly explain some of the differences between these groups. Sparse data bias may have limited the precision of associations between categorical age and the prevalence of hypertension in the HCHS/SOL cohort. We included a continuous measure of age for parsimony and to support the findings. Finally, some acculturation measures within HCHS/SOL (e.g., foreign-born) were limited by low sample size and younger age of US-born HLDB compared to DR-Born HLDB, which might have reduced our statistical power to observe an association of acculturation with prevalence of hypertension. Nevertheless, these limitations don’t invalidate our objective to compare hypertension prevalence and risk factors among Dominicans living in two different social contexts and to report data on the prevalence on hypertension in the context of migration.

## Conclusions

In summary, among two cohorts of Dominican adults living in the US and the DR, we found evidence of similarities and variations in hypertension prevalence and risk factors in both settings, suggesting that context and cultural factors matter among immigrant populations. Regardless of the country of residence, hypertension prevalence among Hispanics/Latinos of Dominican background is high and hypertension control is suboptimal, underscoring the need to improve blood pressure control among this population with country-specific public health strategies. In contrast, while BMI and diabetes were significantly associated with hypertension prevalence, the influence of lifestyle characteristics on disease prevalence varied significantly depending on where Dominican adults primarily lived. These findings were not fully explained by age or sex and warrant further investigation.

## Data Accessibility Statement

This study was based on data from two sources: Dominican Republic’s Cardiology Society (SODOCARDIO for its acronym in Spanish), and Hispanic Community Health Study/Study of Latinos (HCHS/SOL), a United States federally funded population-based study. The datasets generated and/or analyzed during the current study are not publicly available because the availability of the data may be constrained by terms of specific data use agreements and data sharing set forth by the National Institutes of Health (NIH) for HCHS/SOL and SODOCARDIO regulations for ENPREFAR-HAS 17 governing the creation and maintenance of these datasets. All research databases and other information developed in this project can be made available to the scientific community, on reasonable request to the corresponding author and with permission of National Institutes of Health (NIH), HCHS/SOL and SODOCARDIO.

## Additional File

The additional file for this article can be found as follows:

10.5334/gh.1352.s1 Supplementary Files. Supplemental Tables 1 to 6.
