# Supplementary material for: Hypertension Prevalence among Hispanics/Latinos of Dominican Background: A Transnational Comparison of HCHS/SOL and ENPREFAR-HAS-17
Source: Glob Heart. 2024 Aug 26;19(1):71. doi: 10.5334/gh.1352 (PMC11363888; doi:10.5334/gh.1352)
Supplement: Supplementary Files. — Supplemental Tables 1 to 6. [file gh-19-1-1352-s1.pdf]

Table of Contents

Supplemental Table 1: Unadjusted and adjusted prevalence ratios for prevalent hypertension, per ACC/AHA guidelines, among US Dominicans (HCHS/SOL) and DR Dominicans (ENPREFAR-HAS 17)

Supplemental Table 2: Baseline characteristics of the study cohorts, US Dominican from HCHS/SOL (stratified by nativity status) and DR Dominican from ENPREFAR-HAS 17

Supplemental Table 3: Unadjusted and adjusted prevalence ratios for prevalent hypertension, per JNC7 guidelines, among US Dominicans (HCHS/SOL)

Supplemental Table 4: Unadjusted and adjusted prevalence ratios for prevalent hypertension, per ACC/AHA guidelines, among US Dominicans (HCHS/SOL)

Supplemental Table 5: Unadjusted and adjusted prevalence ratios for prevalent hypertension, per JNC7 guidelines, among US Dominicans (HCHS/SOL) and Measures of Acculturation

Supplemental Table 6: Unadjusted and adjusted prevalence ratios for prevalent hypertension, per ACC/AHA guidelines, among US Dominicans (HCHS/SOL) and Measures of Acculturation

Supplemental Table 1: Unadjusted and adjusted prevalence ratios for prevalent hypertension[1], per ACC/AHA guidelines, among US Dominicans (HCHS/SOL[2]) and DR Dominicans (ENPREFAR-HAS 17)

	**US Dominicans from HCHS/SOL[2]**		**DR Dominicans from ENPREFAR-HAS 17**	
	Unadjusted	Sex & Age Adjusted	Unadjusted	Sex & Age Adjusted
	PR (95% CI)	PR (95% CI)	PR (95% CI)	PR (95% CI)
**Age, years[†]**				
18-29	1.0	1.0	1.0	1.0
30-39	2.18 (1.47- 3.24) **	2.25 (1.53- 3.29) ***	1.58 (1.36-1.83) ***	1.33 (1.18-1.49) ***
40-49	2.82 (2.05- 3.88) ***	2.93 (2.15- 4.00) ***	2.04 (1.77-2.34) ***	1.71 (1.53- 1.92) ***
50-59	4.31 (3.23- 5.76) ***	4.37 (3.31- 5.77) ***	2.22 (1.92-2.57) ***	1.47 (1.37- 1.58) ***
60+	5.01 (3.78- 6.64) ***	5.09 (3.86- 6.71) ***	2.59 (2.29-2.93) ***	2.14 (1.94 2.36) ***
**Sex[‡]**				
Male	1.0	1.0	1.0	1.0
Female	0.73 (0.62- 0.86) **	0.72 (0.64- 0.81) ***	0.83 (0.76- 0.89) ***	
**Education level**				
Less than HS	1.0	1.0	1.0	1.0
HS or Equivalent	0.71 (0.57-0.89) **	0.99 (0.81- 1.21) ⁑	0.73 (0.68-0.80) ***	1.04 (0.95- 1.14) ⁑
Greater than HS	0.78 (0.66-0.92) **	1.05 (0.91- 1.22) ⁑	0.60 (0.53-0.67) ***	0.92 (0.81- 1.05) ⁑
**BMI**:				
<25 kg/m^2	1.0	1.0	1.0	1.0
25- 29.9 kg/m^2	2.38 (1.81- 3.13) ***	1.64 (1.28- 2.10) ***	1.37 (1.24- 1.52) ***	1.33 (1.20-1.47) ***
30+ kg/m^2	2.63 (1.97-3.51) ***	2.02 (1.58- 2.59) ***	1.60 (1.45- 1.77) ***	1.62 (1.47- 1.78) ***
**Cigarette Use**				
Non-smoker	1.0	1.0	1.0	1.0
Smoker	0.87 (0.63- 1.20) ⁑	0.88 (0.67- 1.17) ⁑	1.09 (0.97- 1.22) ⁑	1.02 (0.91- 1.14) ⁑
**Alcohol Use**				
Former/ Never	1.0	1.0	1.0	1.0
Current	0.98 (0.84- 1.15) ⁑	1.17 (1.02- 1.34) **	0.80 (0.74- 0.87) ***	0.95 (0.87- 1.03) ⁑
**Diabetes status**				
No Diabetes	1.0	1.0	1.0	1.0
Diabetes	2.11 (1.87- 2.38) ***	1.22 (1.08- 1.38) **	1.60 (1.48- 1.74) ***	1.32 (1.19- 1.46) ***
**Physical Activity**				
Inactive	1.0	1.0	1.0	1.0
Active	0.81 (0.70- 0.95) **	0.91 (0.79- 1.04) ⁑	1.04 (0.96- 1.13) ⁑	1.10 (1.01- 1.19) *
**Health Insurance**				
Uninsured	1.0	1.0	1.0	1.0
Insured	1.32 (1.09- 1.61) **	1.12 (0.95- 1.32) ⁑	1.17 (1.07- 1.29) **	1.03 (0.93- 1.13) ⁑

[†]Not adjusted for age in age/sex adjusted models
[‡]Not adjusted for sex in age/sex adjusted models
[1]ACC/AHA hypertension definition: Systolic blood pressure ≥ 130 mmHg, or diastolic blood pressure ≥ 80 mmHg.
[2]Weighted models
Abbreviations: CI = Confidence Intervals; PR= Prevalence Ratios; HS= High School; BMI= Body Mass Index
P values are reported as follows: *** < 0.05; ** < 0.01; *** < 0.0001; ⁑ > 0.05**

Supplemental Table 2: Baseline characteristics of the study cohorts, US Dominican from HCHS/SOL (stratified by nativity status) and DR Dominican from ENPREFAR-HAS 17

	US Dominicans (HCHS/SOL[3]) DR-Born, N=1305	US Dominicans (HCHS/SOL[3]) US-Born, N=138	DR Dominicans (ENPREFAR-HAS 17) N=2015
Age, years, median IQR (Q1, Q3)	42.1 (29.5, 52.5)	22.7 (19.5, 28.7)	37.0 (26.0, 52.0)
Age (years) categories, n (%)			
18-29	182 (24.1)	110 (77.0)	662 (34.0)
30-39	155 (18.4)	12 (17.2)	420 (21.8)
40-49	358 (24.9)	13 (5.3)	300 (15.4)
50-59	377 (19.2)	3 (0.5)	271 (13.9)
60+	233 (13.4)	0 (0.0)	294 (15.1)
Gender, n (%)			
Female	866 (61.8)	77 (53.7)	1013 (50.3)
Male	439 (38.2)	61 (46.3)	1002 (49.7)
Education level, n (%)			
Less than HS	573 (39.5)	32 (24.6)	569 (28.2)
HS or Equivalent	264 (22.5)	29 (28.3)	1019 (50.6)
Greater than HS	468 (38.0)	77 (47.0)	427 (21.2)
Health insurance, n (%)			
Insured	933 (71.7)	92 (66.8)	1459 (72.4)
Uninsured	319 (28.3)	35 (33.2)	556 (27.6)
Hypertension status (JNC7)[1], n (%)			
Hypertensive	518 (31.0)	10 (3.4)	614 (30.5)
Normotensive	787 (69.0)	128 (96.6)	1401 (69.5)
Hypertension status (ACC/AHA)[2], n (%)			
Hypertensive	730 (48.8)	26 (17.8)	1,113 (55.2)
Normotensive	575 (51.2)	112 (82.2)	902 (44.8)
BMI, kg/m^2, n (%)			
<25	249 (21.3)	44 (28.4)	790 (39.2)
25- 29.9	512 (39.2)	42 (27.4)	668 (33.1)
30+	535 (39.4)	52 (44.2)	553 (27.4)
Diabetes status, n (%)			
No Diabetes	1038 (83.0)	92.9	1886 (93.6)
Diabetes	267 (17.0)	7.1	129 (6.4)
Cigarette use, n (%)			
Non-smoker	1184 (91.4)	73.8	1791 (88.9)
Current	121 (8.6)	26.2	224 (11.1)
Alcohol use, n (%)			
Former/ Never	664 (47.6)	29.7	916 (45.5)
Current	640 (52.4)	70.3	1099 (54.5)
Physical activity, n (%)			
Inactive	272 (19.8)	10.6	1209 (60.0)
Active	1028 (80.2)	120 (89.4)	806 (40.0)

[1] JNC7 hypertension definition: systolic blood pressure ≥ 140 mmHg, or diastolic blood pressure ≥ 90 mmHg

[2] ACC/AHA hypertension definition: Systolic blood pressure ≥ 130 mmHg, or diastolic blood pressure ≥ 80 mmHg.

[3] Weighted statistics. Note: All Ns are unweighted, but HCHS/SOL proportions (%) and means are weighted. Abbreviations: HS= High School; BMI= Body Mass Index

Supplemental Table 3: Unadjusted and adjusted prevalence ratios for prevalent hypertension[1], per JNC7 guidelines, among US Dominicans (HCHS/SOL)

	**US Dominicans from HCHS/SOL[2] BP Harmonization (JNC7)**		**US Dominicans from HCHS/SOL2; Subset - Born in DR Non-Harmonized BP (JNC7)**		**US Dominicans from HCHS/SOL2; Subset - Born in DR Harmonized BP (JNC7)**	
	Unadjusted	Sex & Age Adjusted	Unadjusted	Sex & Age Adjusted	Unadjusted	Sex & Age Adjusted
	PR (95% CI)	PR (95% CI)	PR (95% CI)	PR (95% CI)	PR (95% CI)	PR (95% CI)
**Age, years**†						
18-29	1.0	1.0	1.0	1.0	1.0	1.0
30-39	3.67 (1.81- 7.44) **	3.72 (1.84 – 7.54) **	2.47 (1.17 – 5.22) *	2.65 (1.186 – 5.92) *	2.65 (1.18 – 5.93) *	2.65 (1.19 – 5.92) *
40-49	7.35 (3.71 –14.54) ***	7.50 (3.81 –14.79) ***	4.26 (2.17 – 8.5) ***	5.26 (2.60 – 10.62) ***	5.21 (2.56 – 10.59) ***	5.25 (2.60 – 10.62) ***
50-59	14.32 (7.57-27.10) ***	14.42(7.63-27.24) ***	8.90 (4.61 –17.20) ***	9.91 (5.08 – 19.32) ***	9.94 (5.09 – 19.42) ***	9.91 (5.08 – 19.31) ***
60+	20.16 (10.56-38.48) ***	20.32(10.66–38.73) ***	12.43 (6.45–23.93) ***	14.03 (7.18 –27.39) ***	14.06 (7.17 –27.55) ***	14.03 (7.18 – 27.39) ***
**Gender**‡						
Male	1.0	1.0	1.0	1.0	1.0	1.0
Female	0.86 (0.70 – 1.05) ⁑	0.85 (0.73 – 1.01) ⁑	0.82 (0.68 – 1.00) ⁑	0.85 (0.72 – 1.00) ⁑	0.82 (0.68 – 0.99) *	0.85 (0.72 – 1.00) ⁑
**Education level**						
Less than HS	1.0	1.0	1.0	1.0	1.0	1.0
HS or Equivalent	0.65 (0.52 – 0.83) **	1.06 (0.84 – 1.34) ⁑	0.67 (0.53 – 0.85) **	0.98 (0.78 – 1.23) ⁑	0.66 (0.53 – 0.84) **	0.97 (0.78 – 1.23) ⁑
Greater than HS	0.64 (0.47 – 0.86) **	1.05 (0.82 – 1.36) ⁑	0.74 (0.55 – 0.99) *	1.06 (0.83 – 1.36) ⁑	0.70 (0.51 – 0.95) **	1.06 (0.83 – 1.36) ⁑
**BMI**:						
<25 kg/m^2	1.0	1.0	1.0	1.0	1.0	1.0
25- 29.9 kg/m^2	2.57 (1.80 – 3.67) ***	1.45 (1.05 – 2.00) *	2.09 (1.49 – 2.95) ***	1.46 (1.05 – 2.02) *	2.34 (1.64 – 3.34) ***	1.46 (1.05 – 2.02) *
30+ kg/m^2	3.21 (2.17 – 4.74) ***	2.07 (1.47 – 2.92) ***	2.93 (2.10 – 4.09) ***	2.12 (1.51 – 2.98) ***	3.12 (2.13 – 4.57) ***	2.12 (1.51 – 2.98) ***
**Cigarette Use**						
Non-smoker	1.0	1.0	1.0	1.0	1.0	1.0
Smoker	0.67 (0.45 – 1.0) ⁑	0.69 (0.44 – 1.06) ⁑	0.87 (0.62 – 1.23) ⁑	0.74 (0.47 – 1.16) ⁑	0.89 (0.64 – 1.23) ⁑	0.74 (0.46 – 1.16) ⁑
**Alcohol Use**						
Former/Never	1.0	1.0	1.0	1.0	1.0	1.0
Current	0.77 (0.63 – 0.94) *	1.12 (0.96 – 1.29) ⁑	0.85 (0.71 – 1.02) ⁑	1.14 (0.99 – 1.31) ⁑	0.87 (0.72 – 1.05) ⁑	1.14 (0.99 – 1.31) ⁑
**Diabetes status**						
No Diabetes	1.0	1.0	1.0	1.0	1.0	1.0
Diabetes	3.31 (2.79 – 3.94) ***	1.45 (1.19 – 1.75) **	2.81 (2.39 – 3.31) ***	1.48 (1.24 – 1.77) ***	2.97 (2.54 – 3.47) ***	1.48 (1.24 – 1.77) ***
**Physical Activity**						
Inactive	1.0	1.0	1.0	1.0	1.0	1.0
Active	0.62 (0.51 – 0.75) ***	0.76 (0.63 – 0.91) **	0.70 (0.58 – 0.86) **	0.77 (0.64 – 0.92) **	0.68 (0.56 – 0.82) ***	0.77 (0.64 – 0.92) **
**Health Insurance**						
Uninsured	1.0	1.0	1.0	1.0	1.0	1.0
Insured	1.73 (1.34 – 2.25) ***	1.23 (0.99 – 1.54) ⁑	1.49 (1.17 – 1.90) **	1.21 (0.98 – 1.49) ⁑	1.64 (1.29 – 2.08) ***	1.21 (0.98 – 1.49) ⁑

†Not adjusted for age in age/gender adjusted models

‡Not adjusted for gender in age/gender adjusted models

[1]JNC7 hypertension definition: Systolic blood pressure ≥ 140 mmHg, or diastolic blood pressure ≥ 90 mmHg.

[2]Weighted models

Abbreviations: CI = Confidence Intervals; PR= Prevalence Ratios; HS= High School; BMI= Body Mass Index

P values are reported as follows: *** < 0.05; ** < 0.01; *** < 0.0001; ⁑ > 0.05**

Supplemental Table 4: Unadjusted and adjusted prevalence ratios for prevalent hypertension, per ACC/AHA[1] guidelines, among US Dominicans (HCHS/SOL)

	**US Dominicans from HCHS/SOL[2] BP Harmonization (ACC/AHA[1])**		**US Dominicans from HCHS/SOL2; Subset - Born in DR Non-Harmonized BP (ACC/AHA[1])**		**US Dominicans from HCHS/SOL2; Subset - Born in DR Harmonized BP (ACC/AHA[1])**	
	Unadjusted	Sex & Age Adjusted	Unadjusted	Sex & Age Adjusted	Unadjusted	Sex & Age Adjusted
	PR (95% CI)	PR (95% CI)	PR (95% CI)	PR (95% CI)	PR (95% CI)	PR (95% CI)
**Age, years**†						
18-29	1.0	1.0	1.0	1.0	1.0	1.0
30-39	1.84 (1.22 –2.79) **	1.88 (1.25 – 2.93) **	1.67 (1.14 –2.45) **	1.68 (1.15 – 2.44) *	1.67 (1.14 – 2.45) **	1.67 (1.15 – 2.44) **
40-49	2.57 (1.87 – 3.53) ***	2.65 (1.94 – 3.63) ***	2.22 (1.57–3.14) ***	2.25 (1.59 – 3.16) ***	2.22 (1.57 – 3.14) ***	2.25 (1.59 – 3.16) ***
50-59	3.89 (2.94 – 5.15) ***	3.93 (2.98 – 5.18) ***	3.33 (2.43–4.57) ***	3.32 (2.43 – 4.53) ***	3.33 (2.43 – 4.57) ***	3.32 (2.43 – 4.53) ***
60+	4.38 (3.33 – 5.76) ***	4.43 (3.36 – 5.84) ***	3.75 (2.75–5.11) ***	3.74 (2.75 – 5.09) ***	3.75 (2.75 – 5.11) ***	3.74 (2.74 – 5.09) ***
**Gender**‡						
Male	1.0	1.0	1.0	1.0	1.0	1.0
Female	0.8 (0.69 – 0.93) **	0.79 (0.70 – 0.89) ***	0.78 (0.68 – 0.89) **	0.79 (0.70 – 0.90) **	0.78 (0.68 – 0.89) **	0.79 (0.70 – 0.90) **
**Education level**						
Less than HS	1.0	1.0	1.0	1.0	1.0	1.0
HS or Equivalent	0.82 (0.69 – 0.98) *	1.10 (0.94 – 1.28) ⁑	0.83 (0.70 – 0.98) *	1.05 (0.90 – 1.23) ⁑	0.83 (0.70 – 0.99) *	1.05 (0.90 – 1.23) ⁑
Greater than HS	0.79 (0.62 – 1.00) ⁑	1.07 (0.85 – 1.33) ⁑	0.83 (0.65 – 1.05) ⁑	1.06 (0.86 – 1.32) ⁑	0.83 (0.65 – 1.07) ⁑	1.06 (0.86 – 1.32) ⁑
**BMI**:						
<25 kg/m^2	1.0	1.0	1.0	1.0	1.0	1.0
25- 29.9 kg/m^2	2.22 (0.68 – 2.94) ***	1.56 (1.22 – 2.00) **	1.93 (1.46 –2.56) ***	1.45 (1.12 – 1.87) **	1.93 (1.46 – 2.56) ***	1.45 (1.12 – 1.87) **
30+ kg/m^2	2.74 (2.06 – 3.65) ***	2.13 (1.65 – 2.74) ***	2.63 (1.97 –3.52) ***	2.11 (1.61 – 2.77) ***	2.63 (1.97 – 3.52) ***	2.11 (1.61 – 2.77) ***
**Cigarette Use**						
Non-smoker	1.0	1.0	1.0	1.0	1.0	1.0
Smoker	0.77 (0.56 – 1.07) ⁑	0.78 (0.57 – 1.08) ⁑	0.89 (0.68 – 1.15) ⁑	0.78 (0.57 – 1.09) ⁑	0.89 (0.68 – 1.15) ⁑	0.78 (0.57 – 1.09) ⁑
**Alcohol Use**						
Former/Never	1.0	1.0	1.0	1.0	1.0	1.0
Current	0.95 (0.82 – 1.10) ⁑	1.15 (1.01 – 1.31) *	1.00 (0.86 – 1.15) ⁑	1.12 (0.98 -1.28) ⁑	1.0 (0.86 – 1.15) ⁑	1.12 (0.98 – 1.28) ⁑
**Diabetes status**						
No Diabetes	1.0	1.0	1.0	1.0	1.0	1.0
Diabetes	2.08 (1.84 – 2.34) ***	1.23 (1.08 – 1.41) **	1.93 (1.73 –2.15) ***	1.26 (1.12 – 1.41) ***	1.93 (1.73 – 2.15) ***	1.26 (1.12 – 1.41) ***
**Physical Activity**						
Inactive	1.0	1.0	1.0	1.0	1.0	1.00
Active	0.80 (0.69 – 0.92) **	0.89 (0.78 – 1.02) ⁑	0.86 (0.73 – 1.01) ⁑	0.91 (0.80 – 1.04) ⁑	0.86 (0.73 – 1.01) ⁑	0.91 (0.80 – 1.04) ⁑
**Health Insurance**						
Uninsured	1.0	1.0	1.0	1.0	1.0	1.0
Insured	1.40 (1.14 – 1.72) **	1.18 (0.98 – 1.43) ⁑	1.40 (1.17 – 1.67) **	1.21 (1.02 – 1.44) *	1.40 (1.17 – 1.67) **	1.21 (1.02 – 1.44) *

†Not adjusted for age in age/gender adjusted models ‡Not adjusted for gender in age/gender adjusted models
[1]ACC/AHA hypertension definition: Systolic blood pressure ≥ 130 mmHg, or diastolic blood pressure ≥ 80 mmHg.
[2]Weighted models
Abbreviations: CI = Confidence Intervals; PR= Prevalence Ratios; HS= High School; BMI= Body Mass Index
P values are reported as follows: *** < 0.05; ** < 0.01; *** < 0.0001; ⁑ > 0.05**

Supplemental Table 5: Unadjusted and adjusted prevalence ratios for prevalent hypertension, per JNC7 guidelines, among US Dominicans (HCHS/SOL[1]) and Measures of Acculturation

	US Dominicans from HCHS/SOL[1] Non-Harmonized BP (JNC7[2])		US Dominicans from HCHS/SOL[1] BP Harmonization (JNC7[2])	
	Unadjusted	Sex & Age Adjusted	Unadjusted	Sex & Age Adjusted
	PR (95% CI)	PR (95% CI)	PR (95% CI)	PR (95% CI)
**Language Preference**				
English	1.0	1.0	1.0	1.0
Spanish	3.36 (2.09-5.40) ***	1.17 (0.76-1.78) ⁑	4.29 (2.61-7.07) ***	1.51 (0.96-2.37) ⁑
**Years in the US**				
>10 Years	1.0	1.0	1.0	1.0
<10 Years	0.71 (0.54-0.91) *	1.01 (0.81-1.27) ⁑	0.73 (0.57- 0.94) *	1.05 (0.84-1.32) ⁑
**SASH[†] Language Subscale**				
Below Median Score	1.0	1.0	1.0	1.0
Above Median Score	0.39 (0.30-0.51) ***	0.82 (0.63-1.05) ⁑	0.37 (0.29-0.49) ***	0.78 (0.60-1.01) ⁑
**SASH[†] Social Subscale**				
Below Median Score	1.0	1.0	1.0	1.0
Above Median Score	0.68 (0.57-0.80) ***	0.97 (0.81-1.14) ⁑	0.70 (0.58-0.84) **	1.01 (0.85-1.20) ⁑
**Nativity**				
Born in the US	1.0	1.0	1.0	1.0
Foreign Born	6.31 (3.06-13.0) ***	1.98 (0.97-4.07) ⁑	9.18 (3.82-22.08) ***	2.89 (1.21-6.87) *

[†]Short Acculturation Scale for Hispanics (SASH)
[1]Weighted models
[2]JNC7 hypertension definition: Systolic blood pressure ≥ 140 mmHg, or diastolic blood pressure ≥ 90 mmHg.
[3]ACC/AHA hypertension definition: Systolic blood pressure ≥ 130 mmHg, or diastolic blood pressure ≥ 80 mmHg.
Abbreviations: CI = Confidence Intervals; PR= Prevalence Ratios
P values are reported as follows: *** < 0.05; ** < 0.01; *** < 0.0001; ⁑ > 0.05**

Supplemental Table 6: Unadjusted and adjusted prevalence ratios for prevalent hypertension, per ACC/AHA guidelines among US Dominicans (HCHS/SOL[1]) and Measures of Acculturation

	**US Dominicans from HCHS/SOL[1] Non-Harmonized BP (ACC/AHA[2])**		**US Dominicans from HCHS/SOL[1] BP Harmonization (ACC/AHA[2])**	
	Unadjusted	Sex & Age Adjusted	Unadjusted	Sex & Age Adjusted
	PR (95% CI)	PR (95% CI)	PR (95% CI)	PR (95% CI)
**Language**				
English Preference	1.0	1.0	1.0	1.0
Spanish Preference	2.25 (1.66-3.05) ***	1.22 (0.92- 1.63) ⁑	2.15 (1.60-2.89) ***	1.20 (0.90-1.59) ⁑
**Years in the US**				
>10 Years	1.0	1.0	1.0	1.0
<10 Years	0.71 (0.57-0.89) **	0.88 (0.71- 1.07) ⁑	0.70 (0.56-0.86) **	0.85 (0.70-1.03) ⁑
**SASH[†] Language Subscale**				
Below Median Score	1.0	1.0	1.0	1.0
Above Median Score	0.57 (0.47-0.68) ***	0.90 (0.76-1.07) ⁑	0.55 (0.46-0.66) ***	0.87 (0.73-1.04) ⁑
**SASH[†] Social Subscale**				
Below Median Score	1.0	1.0	1.0	1.0
Above Median Score	0.81 (0.70-0.94) *	1.01 (0.89-1.14) ⁑	0.78 (0.67-0.90) **	0.95 (0.83-1.09) ⁑
**Nativity**				
Born in the US	1.0	1.0	1.0	1.0
Foreign Born	2.54 (1.62- 4.0) ***	1.33 (0.82- 2.15) ⁑	2.74 (1.65- 4.55) ***	1.50 (0.86- 2.59) ⁑

[†]Short Acculturation Scale for Hispanics (SASH)
[1]Weighted models
[2]ACC/AHA hypertension definition: Systolic blood pressure ≥ 130 mmHg, or diastolic blood pressure ≥ 80 mmHg.
Abbreviations: CI = Confidence Intervals; PR= Prevalence Ratios
P values are reported as follows: *** < 0.05; ** < 0.01; *** < 0.0001; ⁑ > 0.05**
